# Power output and fatigue properties using spatially distributed sequential stimulation in a dynamic knee extension task

**DOI:** 10.1007/s00421-017-3675-0

**Published:** 2017-07-03

**Authors:** Marco Laubacher, Anil Efe Aksöz, Robert Riener, Stuart Binder-Macleod, Kenneth J. Hunt

**Affiliations:** 1 Division of Mechanical Engineering, Department of Engineering and Information Technology, Institute for Rehabilitation and Performance Technology, Bern University of Applied Sciences, 3400 Burgdorf, Switzerland; 2 Sensory Motor Systems Laboratory, Department of Health Sciences and Technology, ETH Zurich, Zurich, Switzerland; 3 Department of Physical Therapy, University of Delaware, Newark, USA

**Keywords:** Functional electrical stimulation, Spatially distributed sequential stimulation, Knee dynamometer, Power output, Fatigue

## Abstract

**Purpose:**

The low power output and fatigue resistance during functional electrical stimulation (FES) limits its use for functional applications. The aim of this study was to compare the power output and fatigue properties of spatially distributed sequential stimulation (SDSS) against conventional single electrode stimulation (SES) in an isokinetic knee extension task simulating knee movement during recumbent cycling.

**Methods:**

M. vastus lateralis and m. vastus medialis of eight able-bodied subjects were stimulated for 6 min on both legs with both setups. In the SES setup, target muscles were each stimulated by a pair of electrodes. In SDSS, four small electrodes replaced the SES active electrodes, but reference electrodes were the same. Torque was measured during knee extension movement by a dynamometer at an angular velocity of 110°/s. Mean power (*P*_mean_) was calculated from stimulated extensions for the first 10 extensions, the final 20 extensions and overall. Fatigue is presented as an index, calculated as the decrease with respect to initial power.

**Results:**

*P*_mean_ was significantly higher for SDSS than for SES in the final phase (9.9 ± 4.0 vs. 7.4 ± 4.3 W, *p* = 0.035) and overall (11.5 ± 4.0 vs. 9.2 ± 4.5 W, *p* =  0.037). With SDSS, the reduction in *P*_mean_ was significantly smaller compared to SES (from 14.9 to 9.9 vs. 14.6 to 7.4 W, *p* = 0.024). The absolute mean pulse width was substantially lower with SDSS (62.5 vs. 90.0 µs).

**Conclusion:**

Although less stimulation was applied, SDSS showed a significantly higher mean power output than SES. SDSS also had improved fatigue resistance when compared to conventional stimulation. The SDSS approach may provide substantial performance benefits for cyclical FES applications.

## Introduction

Following spinal cord injury (SCI), mobilisation and exercise play an important role during rehabilitation to prevent and manage the manifold secondary complications of SCI. Functional electrical stimulation (FES) provides one possibility to activate paralysed muscles (Phillips et al. 1998). By applying surface electrodes on the affected muscles, muscle fibres can be stimulated by low levels of pulsed electrical current. Coordinating this stimulation on different muscle groups enables restoration of function, generation of movement patterns and exercise, including the upper and lower-limb cycling systems (Jannsen et al. 1998; Newham and de Donaldson 2007) and FES-rowing (Wheeler et al. 2002). When used regularly over a period of time, FES has also been shown to elicit substantial physiological and health benefits in SCI subjects. Focusing on cycling with SCI subjects, improvements of the cardiopulmonary system (Berry et al. 2008), positive adaptations of the bone mineral density (Frotzler et al. 2008) and increased muscle strength (Duffell et al. 2008) have been observed.

Although much progress has been made in electrical stimulation technology and in its methodology, there are still significant limitations in its performance, especially when applied to SCI subjects to produce a functional movement such as cycling. The maximum power output which can be achieved and the metabolic efficiency are very low, and therefore exercise endurance is limited. While the metabolic efficiency of volitional cycling is around 30%, FES-induced cycling achieves values around 10% (Berry et al. 2012; Glaser et al. 1989; Hunt et al. 2007, 2013; Kjaer et al. 1994). The lack of sensory feedback, for the intra- and inter-muscular coordination of different motor units and the motor-circuit communication to the brainstem, impaired vasomotor response to exercise, reduced vascularisation and a shift of muscle profile towards predominantly fast-fatigable fibre types combined with muscle atrophy might explain the low efficiency (Lavis et al. 2007; Malisoux et al. 2007; Phillips et al. 1998; Pivetta et al. 2014; Takeoka et al. 2014). However, the basic characteristics of artificial stimulation must also play an important role in the low output of FES and cannot be neglected (Maffiuletti 2010).

Natural muscle activation is subtle and complex: it has varying discharge patterns employing non-synchronous, selective recruitment and firing rates where the number of recruited fibres and cross-bridges determine the force produced (Heckman and Enoka 2012; Maladen et al. 2007). In contrast, current FES technology employs a relatively crude approach to muscle stimulation. The muscular power output is mainly increased by changing the stimulation parameters such as frequency, amplitude or pulse width (Baldwin et al. 2006; Gorgey et al. 2006; Gregory et al. 2007) but there are substantial disadvantages and limitations. A general problem with surface stimulation is that motor units of different types are recruited synchronously in a non-selective manner (Jubeau et al. 2007). In addition, particularly at high stimulation intensity, there is only partial recruitment of synergistic motor units and there is co-activation of antagonists (Doucet et al. 2012). With increased pulse duration or amplitude and fixed on/off timing, the muscle activation is more difficult to optimise and the stimulation efficiency drops (Bickel et al. 2011; Gföhler and Lugner 2004; Gregory and Bickel 2005; Hunt et al. 2012). The two most significant limitations of increasing the intensity of stimulation seem to be increased muscular fatigue and patient comfort (Delitto et al. 1992; Lake 1992; Maffiuletti 2010). Addressing fatigue by modulating inter-pulse interval (i.e. lengthening or shortening the interval) or by a simple frequency reduction showed on the one hand increased fatigue resistance, but on the other hand there was decreased overall output power, which in the end is a critical factor for the applicability of such approaches (Binder-Macleod and Guerin 1990; Chou and Binder-Macleod 2007; Gorgey et al. 2009; Graupe et al. 2000; Kesar et al. 2008; Thrasher et al. 2005). By imitating physiological activation through more sophisticated initial stimulation bursts or by increasing both frequency and intensity, statistically significant increases in performance have been observed in isometric measurements (Chou et al. 2008; Cometti et al. 2016). However, both strategies have yet to be evaluated in functional tasks.

With volitionally activated muscles, force is maintained by increasing the firing rate and recruiting more motor units over time (Adam and De Luca 2003; Carpentier et al. 2001; Contessa et al. 2009). This is a challenge for muscle activation through artificial electrical stimulation since electrodes are spatially fixed and the activation of the same fibres results in a drop in force output when they become fatigued (Bickel et al. 2011). Several methods have been investigated to prevent muscular fatigue by distributing the electrodes and stimulating with lower frequencies on each electrode. Synergistic muscles can be stimulated by placing active electrodes on different muscle bellies referred to the same reference electrode and by stimulating with either alternating or cyclical patterns (Decker et al. 2010; Downey et al. 2014; Malesevic et al. 2010; Pournezam et al. 1988). Other studies have focused on the same muscle belly (Laubacher et al. 2016; Nguyen et al. 2011; Sayenko et al. 2014). Both methods showed increased fatigue resistance compared to conventional stimulation, but only the method of Nguyen et al. (2011), which divided one large electrode into four smaller ones, tried to use the full potential of a single muscle belly. They reduced the stimulation frequency from 40 to 10 Hz per electrode and implemented a small time shift between the electrodes: this is termed spatially distributed sequential stimulation, SDSS. The total stimulation frequency thus remained at 40 Hz. This temporally and spatially distributed stimulation gave higher fatigue resistance and showed a more physiological muscle activation in EMG recordings. Since this measurement was a case study performed in an isometric task with a weak relation to a functional movement, it is important that more studies with a functional application are done. The novelty of this study is the length of the protocol and the task-related joint motion (cycling) combined with FES, which allows calculation of the isolated knee extensor power output based on two different stimulation strategies.

The aim of this study was to compare the power output and fatigue properties of spatially distributed sequential stimulation (SDSS) against conventional single electrode stimulation (SES) in an isokinetic knee extension task simulating knee movement during recumbent cycling. This is motivated by the need to make FES-cycling more effective for spinal cord injured patients in their daily life as well as during rehabilitation. We hypothesise that the SDSS setup will produce significantly higher power output and will show a higher fatigue resistance for a 6-min dynamic knee extension task in able-bodied subjects.

## Methods

Eight able-bodied male subjects (age 30.8 ± 3.6 years; height 178.9 ± 10.2 cm; mass 74.8 ± 11.3 kg, mean ± SD) participated in this study. None of the subjects had any known history of neurological or musculoskeletal problems. Each participant gave written informed consent. This study was approved by the local ethics committee (ethics committee of the Swiss Canton of Bern, KEK Bern, Ref.-Nr: KEK-BE. 128/2014).

### Device

**Fig. 1:**
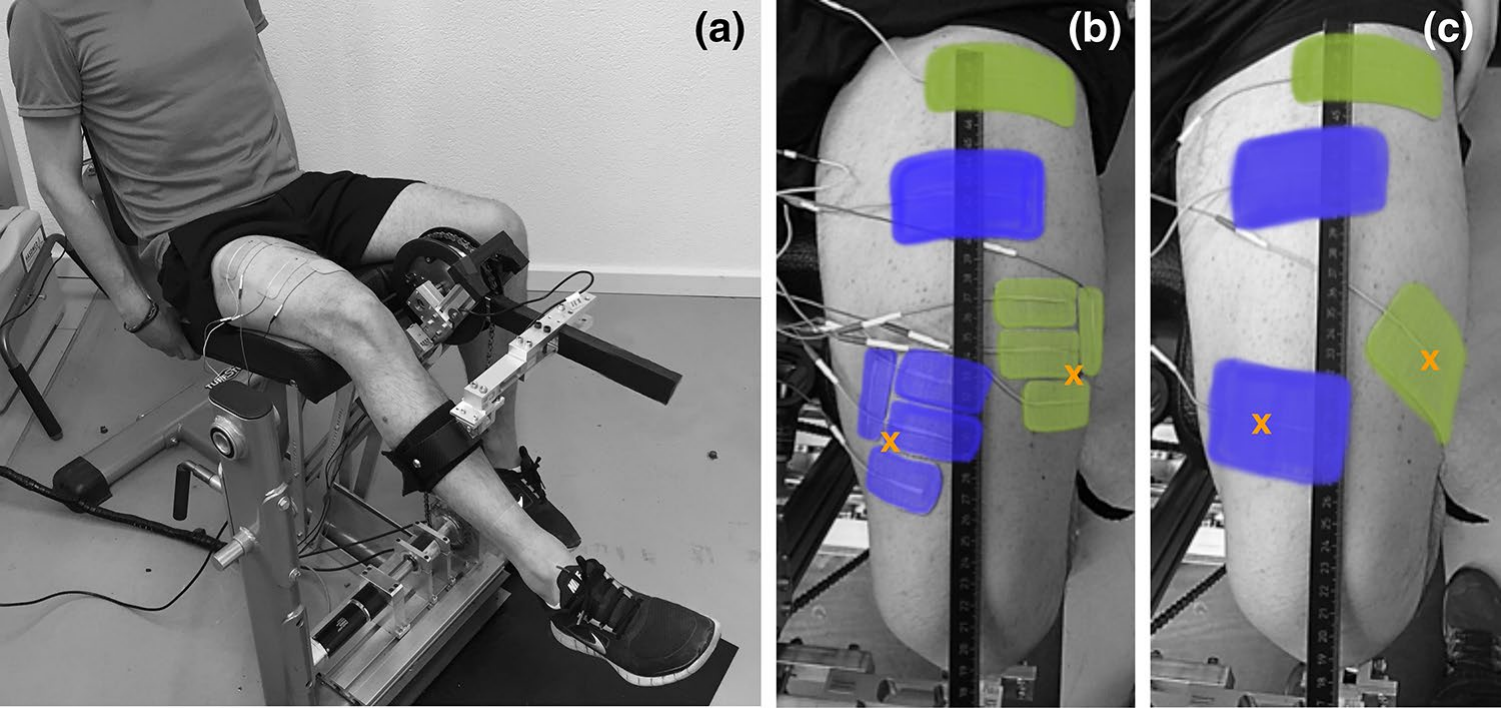
**a** The knee dynamometer measuring the power output of the right leg during stimulation with the SES setup. The leg brace, the lever arm with the load cell and the chain drive system are visible. **b** SDSS setup with the four small electrodes replacing the active electrodes. Electrodes were placed as close as possible to the located motor points. **c** SES setup with two pairs of electrodes. Active electrodes were placed on the motor points of m. vastus medialis and m. vastus lateralis. Motor points are highlighted with an *orange cross* (colour figure online)

A custom-made knee dynamometer (Fig. 1a) capable of moving the leg at a specified velocity and measuring the torque produced during stimulated knee extension was constructed. The system consists of an adjustable rigid mechanical frame and it is able to measure each leg independently, one leg at a time. The lower leg is fixed with a brace to a load cell (LCB130, ME-Meßsysteme GmbH, Germany), and is moved, via lever arm, by a chain drive system connected to a magnetostrictive torque sensor (S-2220-75, NCTE AG Germany). The torque sensor and the load cell are used to bi-directionally measure the effective torque on the gauge bar in real time. Placed on the other side of the torque sensor shaft, a brushless motor (EC45, 250 Watt, Maxon Motor AG, Switzerland) is used with a planetary gear head (Gear Ratio: 156:1, GP42, Maxon Motor AG, Switzerland) to produce the desired isokinetic motion. The actuator can generate a maximum continuous output torque of 90 Nm. A position sensor (Vert-X 28 Analog Position Sensor, Contelec Gmbh, Switzerland) is used for the angle measurement with a resolution of 0.648°.

The angular range of motion at the knee joint was 35°–130° (180° means straight leg) and the measurements were performed at a mean angular velocity of ~110°/s at the knee joint, which is equivalent to a cycling cadence of 50 rpm. Matlab/Simulink and the Real-Time Toolbox (Mathworks Inc., USA) were used for device control and data acquisition. A graphical user interface was implemented for setting up and controlling the device, the stimulation parameters and the timing.

### Stimulation

Two different electrode setups were compared: SES and SDSS. Subjects were stimulated in both setups with rectangular bi-phasic pulses of constant amplitude of 40 mA. Those electrical pulses were generated under PC control with an eight-channel stimulator (RehaStim, Hasomed Gmbh, Germany) with a current range of 0–126 mA (2 mA steps), a pulse width range of 0–500 µs (1 µs steps) and a frequency range of 0–100 Hz. One muscle motor point was detected for each stimulated muscle prior to measurement with a stimulation pen (Motor Point Pen, Compex SA, Switzerland). The skin was cleaned and the body hair shaved at the position of the electrodes. In the SES setup, self-adhesive electrodes with a dimension of 9 × 5 cm (Pals Platinum, Axelgaard Mfg. Co., LTD, USA) were placed on the motor points of the m. vastus lateralis and medialis and reference electrodes with the same size were placed 10–15 cm proximal of the corresponding muscle motor point (Fig. 1b). The term SES is hereby related to the single pair of electrodes per activated muscle and not to the muscle group. The frequency was 35 Hz and the stimulation was applied only during the knee-extension phase of the motion, over a knee-angle range of 55°–115°. In the SDSS setup (Fig. 1c), the active electrodes were implemented as four small electrodes each with a size of 4.5 × 2.5 cm, located around the previously detected motor point. Each of the four electrodes stimulated with a frequency of 8.75 Hz and a phase shift of 90°, which in sum corresponds to the overall stimulation frequency of the SES setup of 35 Hz. The reference electrodes and the stimulation angle did not change and remained the same as for the SES setup. In each session, the pulse width was adapted to the subject according to the familiarisation detailed below. For this study, the mean pulse width applied was 90.0 ± 17.7 µs for SES and 62.5 ± 13.8 µs for SDSS.

### Procedure

Each subject participated in two sessions and in each session two measurements were conducted, with one measurement for each leg. Between the two independent leg measurements, subjects had a break of 15 min. Stimulation order (SES then SDSS vs. SDSS then SES) was chosen randomly. Before each measurement (leg and setup), a familiarisation was conducted. Subjects were placed on the dynamometer system and individual adjustments to body proportions were made. Then a 2-min passive phase was started where the measured leg was moved by the device without stimulation (non-stimulation phase, ns-phase). This was used as a baseline measurement for the leg movement resistance. Then the pulse width was manually increased after every third extension, starting at 0 µs. Pulse width was increased up to the subject’s pain tolerance level. 80% of the observed maximal tolerated pulse width (PW_max_) was then used for the following test measurement.

After a rest period of about 10 min following familiarisation, the measurement started with an ns-phase of 2 min and then a stimulation phase (st-phase) of 6 min followed. After that a second 2-min ns-phase completed the measurement. Each session was conducted on a different day with at least one day of rest in between. Motor points and electrode positions were marked to ensure identical placement across the sessions.

### Outcomes and statistical analysis

**Fig. 2:**
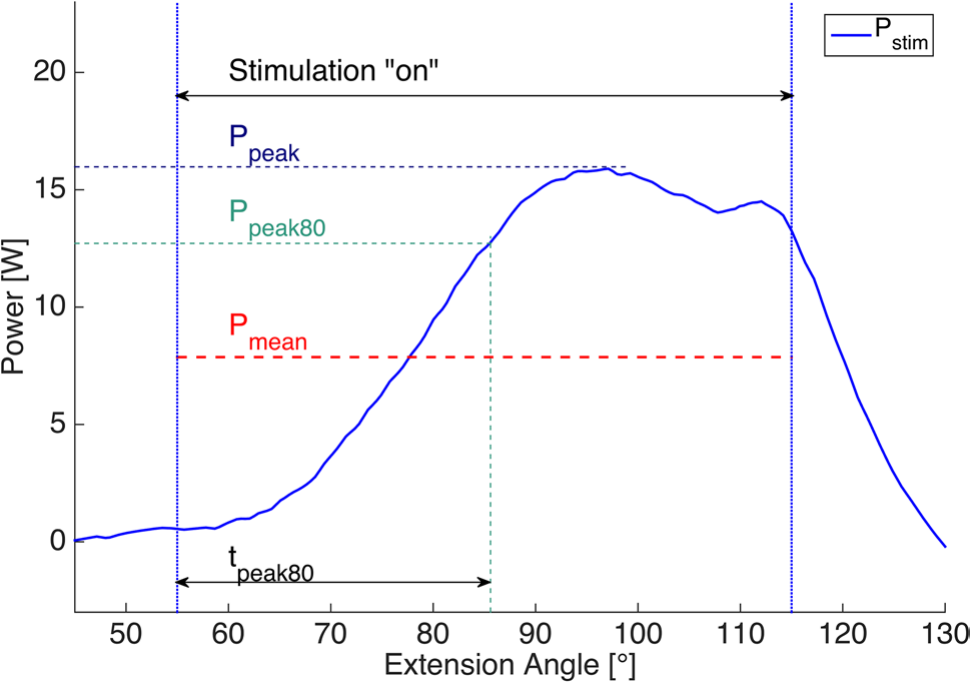
The curve represents the power output of one stimulated leg extension (*P*_stim_) with its characterising output parameters

Only the stimulated extension phase of the knee joint motion was evaluated. The measured torque together with the angular speed was used to calculate the gross output power *P*_m_. The power used to move the leg during ns-phase was defined as *P*_ns_. The effective power output of one stimulation cycle, *P*_stim_, is then obtained as *P*_stim_ = *P*_m_ − *P*_ns_. For each measurement, the following parameters were calculated (Fig. 2): (a) mean power output over the stimulation angle range during one extension (*P*_mean_), (b) peak power output (*P*_peak_) and (c) the time from onset of the stimulation to 80% of *P*_peak_ (*t*_peak80_). To allow comparison between the different stimulation strategies and their efficiency, the differing pulse widths used were scaled to an input pulse width of 100 µs (*P*_stim,s_ and *P*_peak,s_, respectively). For example, if *P*_mean_ is the mean power output of subject A that reached with a stimulation pulse width of 80 µs, then the mean scaled power output (*P*_mean,s_) of that subject is *P*_mean_ × (100/80). The output parameters (a) and (b), regular and scaled, and (c) are presented as means ± standard deviations and were calculated for the initial 10 stimulated extensions, the final 20 extensions and overall (200 extensions).

A fatigue index (FI) based on *P*_mean_ describes the loss of power between the ten initial knee extensions (*P*_init_) and the final 20 knee extensions (*P*_final_) from the stimulated phase. Thus, FI = 1 − (*P*_init_ − *P*_final_)/*P*_init_. The higher the value, the higher the fatigue resistance; FI = 1 means no fatigue.

Data from the left and right legs were averaged for each subject. The differences between SES and SDSS for each outcome variable were tested for normality using the Shapiro–Wilk test, and then a paired *t* test for normally distributed data and a Wilcoxon test for non-normally distributed data were applied to test for any significant differences of means. The significance level was set at *α* = 0.05 for all tests. Statistical analyses were carried out using the Matlab Statistics and Machine Learning Toolbox (Mathworks Inc., USA).

## Results

**Fig. 3:**
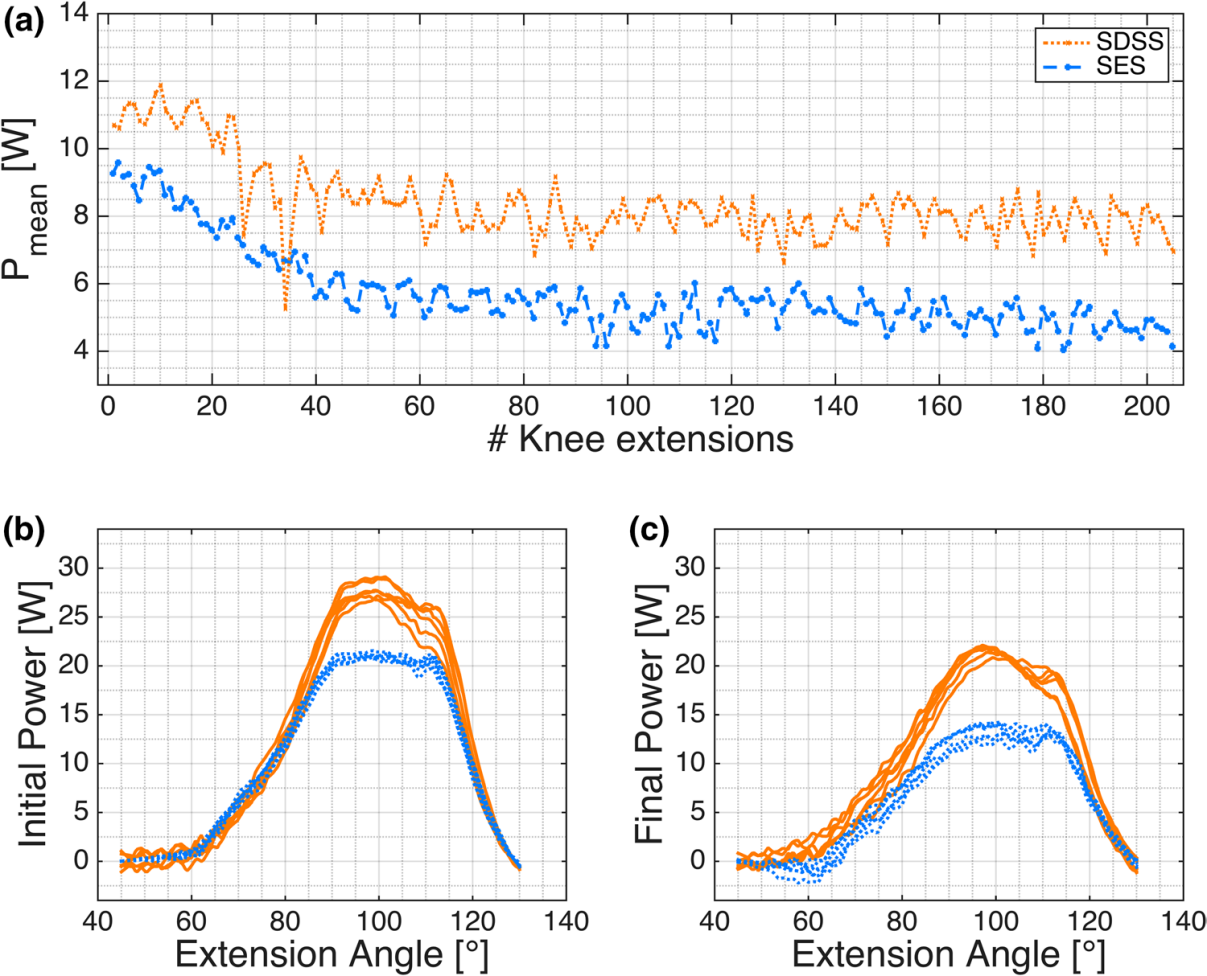
**a** Power output (*P*_mean_) during the 6-min stimulated knee extension of one subject’s right leg. **b**, **c** The corresponding power curves of six consecutive stimulated knee extensions (*P*_stim_) of the same subject during the initial phase (**b**) and during the final phase (**c**)

**Fig. 4:**
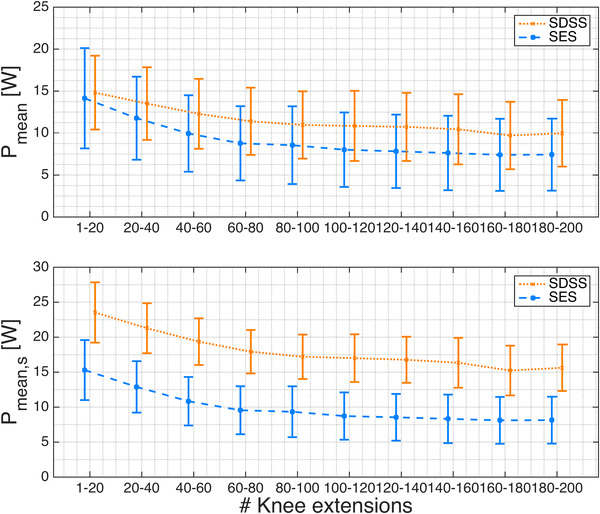
**a** Power output (*P*_mean_) during the 6-min stimulated knee extension and **b** scaled power output (*P*_mean,s_) during the 6-min stimulated knee extension, with *P*_stim_ scaled to an input pulse width of 100 µs. The *circles* represent the mean of 20 consecutive knee extensions. The *error bars* show the standard deviations

**Fig. 5:**
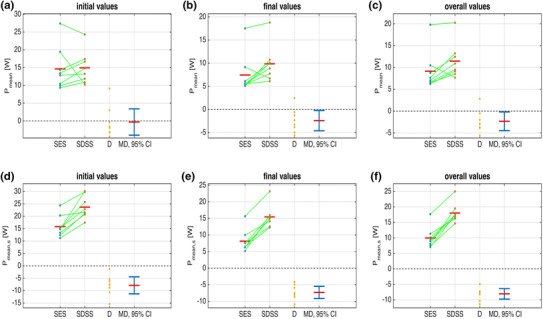
Data samples for *P*_mean_ (**a**–**c**) and *P*_mean,s_ (**d**–**f**) for the initial (**a**, **d**), the final (**b**, **e**) and the overall (**c**, **f**) stimulation phases for both setups; the *green lines* link the sample pairs from each subject; the *red bars* depict mean values. *D* is the difference between the paired samples. MD is the mean difference (*red bar*) with its 95% confidence interval (CI) in *blue*. Inclusion of the value 0 within the 95% CI in **a** signifies a non-significant difference in this case, conforming with *p* > 0.05 (Table 1). For all other tests, **b**–**f**, the value 0 lies outwith the CI, thus signifying significant differences between SES and SDSS with *p* < 0.05 (Table 1) (colour figure online)

The time courses of *P*_stim_ and *P*_stim,s_ show a slower decrease and flatten out later at a higher level in the SDSS setup compared to the SES setup (Figs. 3a, 4a, b). The initial phase revealed no difference for SDSS vs. SES for *P*_mean_ (14.9 ± 4.6 vs. 14.6 ± 6.1 W (mean ± SD), *p* = 0.85, Fig. 5a), whereas *P*_mean,s_ for SDSS was significantly higher (23.7 ± 4.5 vs. 15.8 ± 4.4 W, *p* = 0.001, Fig. 5d). *P*_mean_ and *P*_mean,s_ were both significantly higher for SDSS than for SES in the final phase (9.9 ± 4.0 vs. 7.4 ± 4.3 W, *p* = 0.035, and 15.4 ± 3.4 vs. 8.1 ± 3.3 W, *p* < 0.0001, respectively, Figs. 3c, 5b, e), and in the overall calculation, both were significantly higher for SDSS than for SES (11.5 ± 4.0 vs. 9.2 ± 4.5 W, *p* = 0.037 and 18.0 ± 3.1 vs. 10.0 ± 3.4 W, *p* < 0.0001, respectively, Fig. 5c, f). In contrast to SDSS, where the power dropped on average by 34% (from 14.9 to 9.9 W; Fig. 5a, b; Table 1), SES showed a significantly lower fatigue resistance (fatigue index 0.67 ± 0.13 vs. 0.51 ± 0.10, SDSS vs. SES, *p* = 0.024); with SES, power dropped by 49% (from 14.6 to 7.4 W; Fig. 5a, b; Table 1). No significant differences in any stimulation phase were found for *t*_peak80_ (Table 1). For calculation of *P*_mean,s_, the scaling factor for SDSS was 1.6 and for SES it was 1.1, reflecting the substantially lower absolute mean stimulation intensity used with SDSS (62.5 vs. 90.0 µs, SDSS vs. SES). All sample differences showed a normal distribution, except for the fatigue resistance data; the primary outcome measures and hypothesis test results are summarised in Table 1.

**Table 1:** Primary outcome measures for paired comparisons and *p* values (*α* = 0.05) for comparison of means (*n* = 8)

Phase	Parameter	Mean ± SD		MD (95% CI)	*p* value
		SES	SDSS		
Initial	*P*_mean_ (W)	14.6 ± 6.1	14.9 ± 4.6	−0.3 (−4.0, 3.4)	0.85
	*P*_mean,s_ (W)	15.8 ± 4.4	23.7 ± 4.5	−7.9 (−11.3, −4.4)	0.0010
	*P*_peak_ (W)	31.2 ± 12.6	31.7 ± 9.6	−0.5 (−7.9, 6.9)	0.88
	*P*_peak,s_ (W)	33.8 ± 9.4	50.4 ± 10.3	−16.6 (−24.0, −9.2)	0.0011
	*t*_peak80_ (ms)	359.4 ± 54.7	348.9 ± 32.4	10.6 (−29.7, 50.8)	0.55
Final	*P*_mean_ (W)	7.4 ± 4.3	9.9 ± 4.0	−2.4 (−5.6, −0.2)	0.035
	*P*_mean,s_ (W)	8.1 ± 3.3	15.4 ± 3.4	−7.3 (−9.1, −5.5)	<0.0001
	*P*_peak_ (W)	16.0 ± 8.0	21.4 ± 8.4	−5.4 (−9.6, −1.1)	0.021
	*P*_peak,s_ (W)	17.6 ± 6.0	33.5 ± 7.6	−16.0 (−20.6, −11.3)	<0.0001
	*t*_peak80_ (ms)	364.1 ± 58.6	363.8 ± 19.7	0.3 (−42.1, 42.7)	0.25
Overall	*P*_mean_ (W)	9.2 ± 4.5	11.5 ± 4.0	−2.3 (−4.5, −0.2)	0.037
	*P*_mean,s_ (W)	10.0 ± 3.4	18.0 ± 3.1	−8.1 (−9.8, −6.4)	<0.0001
	*P*_peak_ (W)	19.4 ± 8.7	24.6 ± 8.5	−5.2 (−9.2, −1.3)	0.017
	*P*_peak,s_ (W)	21.2 ± 6.4	38.8 ± 6.9	−17.6 (−21.4, −13.8)	<0.0001
	*t*_peak80_ (ms)	359.5 ± 43.7	359.2 ± 16.1	0.3 (−38.6, 39.3)	0.55
Fatigue index		0.51 ± 0.10	0.67 ± 0.13	−0.16 (−0.29, −0.03)	0.024
Pulse width (µs)		90.0 ± 17.7	62.5 ± 13.8	−27.5 (−34.7, −20.4)	<0.0001

All *p* values were calculated with a paired *t* test, except fatigue index where a Wilcoxon test for non-normally distributed data was applied

*SES* single electrode stimulation, *SDSS* spatially distributed sequential stimulation, *MD* mean difference, *SD* standard deviation, *CI* confidence interval

## Discussion

The aim of this study was to compare the power output and fatigue properties of spatially distributed sequential stimulation (SDSS) against conventional single electrode stimulation (SES) in an isokinetic knee extension task simulating knee movement during recumbent cycling.

Although less stimulation was applied, the SDSS setup showed a significantly higher power output *P*_mean_ overall as well as during the final 20 extensions. The initial power output was not significantly different. Scaling the power output using pulse width, *P*_mean,s_ showed substantially larger differences and significance levels during the initial and final phases and overall, which highlights its significantly higher efficiency. The SDSS setup was significantly more fatigue resistant than the conventional SES stimulation setup.

### Power output development

Looking at the time course of *P*_mean_ (Fig. 3a), the highest power output produced in both setups was reached during the first ten extensions. The muscles are not yet fatigued and it can be assumed that *P*_mean_ in the initial phase is the maximum possible tolerated power output for the corresponding electrode setup and muscle group (Bickel et al. 2011). Although subjects tolerated a higher pulse width during SES, *P*_mean_ was lower, but not significantly, in the initial phase. All subjects used significantly lower pulse widths during SDSS and this might lead to the expectation of higher maximal power outputs for SES, given that an increasing pulse width usually corresponds to increased power output at a constant frequency (Baldwin et al. 2006; Gregory et al. 2007). Finding no significant differences during the initial extensions indicates that both setups recruit and activate, in sum, a similar number of motor units during one movement cycle (Hodson-Tole and Wakeling 2009).

Considering the power output development over the 6 min of stimulation, the two setups were performed completely in a different manner. With SES, *P*_mean_ dropped by a third in the first 60 extensions and further decreased to 50% of the initial power output. In contrast, *P*_mean_ decreased in the SDSS setup much more slowly during the first 60 extensions and flattened out towards the end of the stimulation phase at a level of about 66% of the initial *P*_mean_ (Figs. 3a, 4). This better fatigue resistance confirms previous observations from Nguyen et al. (2011), Sayenko et al. (2014) and Popovic and Malesevic (2009). With a group of healthy subjects, Sayenko et al. (2014) observed bigger differences when focusing on the activation curve and the fatigue of the m. soleus. In contrast to those studies, the protocol used in the present study is three times longer and the m. quadriceps is stimulated during a concentric dynamic movement.

The separation of one large electrode with 35 Hz stimulation frequency into four small electrodes, each stimulating with a much lower frequency of 8.75 Hz per electrode, seems to have many benefits. The influence of stimulation frequency on fatigue has been investigated by many other investigators. It has been shown that low frequencies have lower ATP costs per contraction (Bergstrom and Hultman 1988; Fitts 1994), and thus are more efficient in binding cross-bridges. Additionally, an increase in inorganic phosphate and pH factors (Russ et al. 2002) and problems in Ca^2+^ release at higher frequencies are factors that cause muscle fatigue (Westerblad et al. 1990, 2000). On the one hand, it can be held that increasing the frequency accentuates muscle fatigue while decreasing the frequency reduces muscle fatigue. On the other hand, the lower frequency is usually linked with a decrease in power output (Binder-Macleod and Guerin 1990; Chou et al. 2008; Chou and Binder-Macleod 2007; Dreibati et al. 2010; Gorgey et al. 2009; Kesar et al. 2008), which can be disadvantageous for functional tasks.

Based on previous publications (Nguyen et al. 2011; Sayenko et al. 2014), we expected a higher fatigue resistance, but not necessarily the significantly higher power output with the SDSS setup. Although lower pulse widths and lower frequencies were applied on a single SDSS electrode, all except two subjects showed a higher *P*_mean_ in the initial phase with SDSS and just one of them stayed lower with SES in the final phase (Figs. 3b, c, 5a, b). Low pulse width combined with low frequencies is usually directly linked with a decreased power output (Baldwin et al. 2006; Gorgey et al. 2006, 2009), so the significant differences (viz. higher power output with SDSS) in our measurements can not be due only to frequency, but must result from the combination of the spatially and sequentially distributed electrodes. Sayenko et al. (2014) found with EMG measurements partial activation of different parts of the stimulated muscle depending on the placement of the small electrodes. This supports the theory that different motor units are stimulated with the different sub-electrodes and they are allowed more time to recover between subsequent activations.

The low frequency of 8.75 Hz is sufficient to activate muscle fibres in the m. quadriceps (Fig. 3) but fibres activated around 10 Hz would not be expected to generate high forces (Roos et al. 1999; Wessberg and Kakuda 1999). So, how can the higher power output of the SDSS setup be explained? During voluntary contractions, force is increased by recruiting more motor units and increased cross-bridge bindings, based on increased firing rates (Bellemare et al. 1983; Roos et al. 1999; Rubinstein and Kamen 2005). Here, stimulation with SDSS leads to a higher current density on specific points on the muscle (Kuhn et al. 2010) but since the small electrodes are placed quite close to each other, the generated electrical field is assumed to be overlaid in some muscular parts, thus some motor neurons may still be stimulated at 35 Hz. This summation of different action potentials might be one mechanism to increase the number of cross-bridges and, accordingly, the produced force compared to the force produced by the lower density currents of the larger active electrode. The higher force might also be explained in part by increased intramuscular coordination with more and different motor units involved in the contraction cycle. The mechanism in SDSS whereby the electric field is changed constantly (phase shift together with the spatial shift) might activate other neural circuits, which again activate some other muscle parts in the same muscle group. This complementary activation of different parts of the stimulated muscle results in a stronger total muscle contraction and less fatigue (Fig. 3). This is comparable to a voluntary contraction, where neuromuscular circuits with motor unit inhibitions and low firing rates, together with phase shifts, provide smooth contractions (Broman et al. 1985; De Luca et al. 1982).

### Methodology/scaling/electrode setting

A familiarisation session was used to define stimulation tolerance and parameters. Based on individual tolerance levels, soft tissue and muscle constitution, each subject and leg needs its own specific stimulation parameters (Keller and Kuhn 2008). It can be assumed that an approximately linear relationship exists between stimulation intensity and force production at moderate stimulation levels. By stimulating here at 80% of the individual tolerance level, the aim was to remain in this linear phase (Adam and De Luca 2003; Bickel et al. 2004; Hillegass and Dudley 1999). Our primary strategy in this study was to compare the two different electrode setups and we tried to change as few of the other parameters as possible to reduce confounding factors. The basic stimulation frequency was chosen here to be 35 Hz, which is known to be a good trade-off between fatigue resistance and force generation (Hunt et al. 2012). Changing pulse width and keeping pulse amplitude constant at 40 mA means that the difference in current density between SES and SDSS stayed the same for all subjects. It would have been possible to stimulate with lower amplitude and longer pulse widths, but since amplitude together with the electrode size is mainly responsible for the current density (Alon et al. 1994), it might have been that the more different reaction of the subjects to wide pulse width, high frequency stimulation as investigated by Wegrzyk et al. (2015) would have influenced the results more than the different electrode setup (see low occurrence of responders (40%) in Wegrzyk et al. (2015)). The maximal tolerable stimulation should be used but without influencing the movement. Post hoc, transferring the upper pain level to a numeric pain rating scale (1–10) (McCaffery and Beebe 1994), the familiarisation was stopped at the level of approximately 6–7 for each subject. The pain level during measurement would not have exceeded level 5 (moderate pain), which does not interfere with movement. This was asked during the measurement but without referring to a pain scale. Therefore, the generated power output in this study is always related to 80% of the maximal tolerated stimulation intensity. The lower mean pulse width found with the SDSS electrode configuration shows that this setup is generally more painful for able-bodied subjects. This is in line with previous observations by Kuhn et al. (2010), where smaller electrodes caused more pain. The variation of pulse widths among the subjects reflects variations in soft tissue composition and pain tolerance.

The scaled power output takes account of the different pulse widths and normalises the stimulation intensity between the setups. Scaling the input pulse widths to a notional 100 µs highlights the differences between the two setups at equal inputs and provides values to compare the efficiency of the two setups. *P*_mean,s_ obtained with the SDSS electrode configuration showed substantially larger differences and significance levels during the initial and final phases and overall, which emphasises the higher efficiency of the SDSS setup compared to the SES configuration.

Nguyen et al. (2011) and Sayenko et al. (2014) used a symmetrical arrangement for the SDSS setup, where the electrodes covered exactly the same surface as in SES. In the present study, both setups covered the same skin area, but over a slightly different part of the stimulated muscle. SDSS electrode positioning was chosen dependent on the prior motor point (MP) detection and on the size and shape of the muscle (Fig. 1c). This is because the goal of the positioning was to be as close as possible to the MP and to cover the stimulating muscle as well as possible to optimise the power output (Gobbo et al. 2014; Maffiuletti 2010).

This study showed some major benefits of the SDSS setup compared to SES regarding fatigue resistance and power output but the study has some limitations which are discussed here. One limitation is that the measurements were conducted with able-bodied subjects, where the influence of volitional movement cannot be fully excluded. The stimulation intensity was based on subjective and individual pain tolerance, obtained informally from each subject for each leg and pattern during familiarisation. For better uniformity and comparability among the subjects, an established pain scale should be used in future studies. A further limitation is that, while the dynamometer provided a good basis for assessment of a dynamic knee extension task, it is still a simplification of a real cycling movement. The influence of hip flexion and the coordinated activation of the hamstrings were not considered in the dynamometer setup. The experimental setup used, together with the applied stimulation parameters, is just one possibility and the results obtained are strongly linked with these configurations. The influence of changing pulse width, amplitude and/or frequency in conjunction with specific electrode configurations is a further subject for future research studies.

## Conclusions

Although less stimulation was applied, SDSS showed a significantly higher mean power output than SES and also had improved fatigue resistance when compared to conventional stimulation. The present study confirms the benefits of SDSS, as previously observed by Nguyen et al. (2011) and Sayenko et al. (2014), and expands the application to a dynamic knee extension task with able-bodied subjects. The SDSS approach may therefore provide substantial performance benefits for cyclical FES applications.

The positive results suggest the need for further investigations where other approaches to modulation of stimulation parameters are combined with SDSS; it was shown that varying stimulation parameters can increase fatigue resistance and/or generated force (Doucet and Griffin 2012; Downey et al. 2011; Maladen et al. 2007; Slade et al. 2003), and this outcome might be strengthened when SDSS is concurrently applied. Another option would be to develop more sophisticated electrodes and stimulators for more complex movements and to have more possibilities to vary the spatial component. New strategies can then be developed and other challenges such as closed-loop control can be faced (Downey et al. 2015). Also, the benefits of the SDSS approach need to be validated in target patient populations most likely to benefit from FES, such as spinal cord-injured subjects (Scott et al. 2007).

## Abbreviations

EMG: Electromyography
FES: Functional electrical stimulation
Final: The last 20 stimulated knee extensions
Initial: The first 10 stimulated knee extensions
MVC: Maximal voluntary contraction
ns-phase: Non-stimulation phase
Overall: All stimulated knee extensions
*P*_m_: Gross power output
*P*_mean_: Mean power output during stimulated leg extension
*P*_mean,s_: Scaled mean power output during stimulated leg extension
*P*_ns_: Power used to move the leg during the non-stimulation phase
*P*_peak_: Peak power output during stimulated leg extension
*P*_peak,s_: Scaled peak power output during stimulated leg extension
*P*_stim_: Net power output during stimulation = *P*_m_ − *P*_ns_
*P*_stim,s_: Scaled net power output during stimulation
PW_max_: Maximal tolerated pulse width
SCI: Spinal cord injury
SDSS: Spatially distributed sequential stimulation
SES: Single electrode stimulation
st-phase: Stimulation phase
*t*_peak80_: Time from onset of the stimulation to 80% of *P*_peak_
